# Tobacco and Alcohol Content in Top Vietnamese YouTube Music Videos: Content Analysis

**DOI:** 10.2196/55555

**Published:** 2024-11-08

**Authors:** Thi Phuong Thao Tran, Thu Trang Vu, Yachao Li, Lucy Popova

**Affiliations:** 1 School of Public Health, Georgia State University Atlanta, GA United States; 2 Hanoi University of Public Health Hanoi Vietnam; 3 The College of New Jersey Ewing Township, NJ United States

**Keywords:** risk, risk factor, tobacco content, alcohol content, tobacco, alcohol, tobacco portrayal, alcohol portrayal, music video, Vietnam, Vietnamese, YouTube, social media, socials, youth, adolescent, teen, teenager, young adult

## Abstract

**Background:**

Seeing portrayals of tobacco and alcohol in music videos (MVs) may reduce perceived risks, increase susceptibility, and lead to the initiation of tobacco and alcohol use among adolescents and young adults. Previous studies have predominantly concentrated on assessing tobacco and alcohol contents in English-language MVs within Western countries. However, many other countries have not only been influenced by the English music market but have also produced music in their native languages, and this content remains underexamined.

**Objective:**

This study aims to investigate the prevalence of tobacco- and alcohol-related content in top Vietnamese MVs on YouTube from 2013 to 2021, to describe how tobacco and alcohol are portrayed in these MVs, and to examine associations between these portrayals and MV characteristics.

**Methods:**

A total of 410 Vietnamese MVs, including the top 40 or 50 most viewed released each year between 2013 and 2021, were analyzed. General information, such as the song name, its release date and ranking, age restriction, musical genre, and type of MV, was collected. We examined tobacco and alcohol content in the MVs, with specific details such as tobacco types, their brands, as well as the number, age, sex, and roles of individuals smoking or drinking.

**Results:**

Among the 410 MVs, 36 (8.8%) contained tobacco-related content and 136 (33.2%) featured alcohol-related content. Additionally, 28 (6.8%) out of 410 MVs included both tobacco and alcohol content. The prevalence of videos with tobacco and alcohol content fluctuated over the years. In MVs with tobacco-related content, a higher proportion of hip-hop or rap songs contained tobacco-related content (n=6, 30%) compared to other music genres. In MVs with tobacco-related content, cigarettes were the most frequently shown product (n=28, 77.8%), and smoking scenes were often depicted at parties (n=13, 36.1%) and during dancing and singing scenes (n=12, 33.3%). Among the 31 MVs portraying actual tobacco use, tobacco use was typically depicted with 1 person, often a young adult male, while 38.7% (n=12) showed singer(s) smoking. For MVs with alcohol-related content, there was a high proportion showing alcohol images at parties, bars, or pubs (n=96, 70.6%). Among 87 MVs containing drinking scenes, 60.9% (n=53) involved groups of young adults of both sexes, and 64% (n=56) depicted singers drinking. Additionally, only 2 (5.6%) MVs included health warnings about tobacco harm, and 2 MVs (1.5%) included warnings about drinking restricted to individuals 18 years and above.

**Conclusions:**

The notable prevalence of tobacco and alcohol content in leading Vietnamese YouTube MVs raises concerns, especially as most of this content is portrayed without any warnings. The study underscores a regulatory gap in addressing such content on the internet, emphasizing the urgent need for stricter regulations and age restrictions on platforms such as YouTube.

## Introduction

The tobacco and alcohol industry has invested billions of dollars in a wide range of direct and indirect advertising, promotion, and sponsorship (APS) tactics to attract people worldwide to purchase its products [1,2]. These tactics encompass public entertainment, sponsoring sports events, advertising on radio, television, magazines, newspapers, point-of-sale, and other forms such as distributing free samples, loyalty schemes, and coupons. The negative impacts of APS tactics have been well documented, including smoking and drinking initiation [3-6], higher levels of tobacco and alcohol consumption [4,7-9], and the fostering of positive attitudes or misunderstandings about tobacco and alcohol use [10-12]. Therefore, prohibiting APS is a key element of tobacco control and one of the “best buys” for reducing the burden of harmful alcohol use [13,14].

However, advertising has extended well beyond traditional media in recent years thanks to the borderless growth of digital marketing [15,16]. With the rapid expansion of social media platforms, the tobacco and alcohol industries have increasingly leveraged them as powerful marketing tools, often through partnerships with celebrities and influencers to promote their products [17,18]. YouTube, the largest video-sharing platform and most popular social media among young people (used by 95% of teens in the United States), has served as an effective marketing medium through its music videos (MVs) [17,19]. For instance, in England, YouTube MVs delivered approximately 1006 million gross alcohol impressions (95% CI 748-1264) and 203 million tobacco impressions (95% CI 151- 255) to the British population in 2016 [20]. Alcohol- and tobacco-related imagery and lyrics have been blended into numerous MVs, indirectly and subtly promoting these unhealthy behaviors. A prior study found that some MVs overtly encouraged excessive drinking and drunkenness without showing any negative consequences to the drinker [21]. As a result, watching these MVs, particularly those with positive alcohol portrayals, is linked to adolescent drinking initiation and increased drinking [22].

Previous studies have primarily examined tobacco and alcohol content in English-language MVs within Western countries. However, many other countries have not only been influenced by the English music market but have also created music in their native languages, often accompanied by MVs. There is a lack of research on the prevalence of tobacco and alcohol content and the portrayal of related features in MVs in non-English languages [23].

In Vietnam, there are approximately 65 million social media users, with YouTube reigning supreme as the dominant platform for sharing MVs [24]. YouTube is also the second most widely used social media platform after Facebook, particularly among children, adolescents, and young adults who consider it their primary choice for music consumption [24]. Vietnam ranks among the top 10 countries worldwide in cigarette consumption, with approximately 15.6 million smokers [25]. It also ranks among the top 3 countries worldwide in terms of alcohol per capita consumption [26]. Given these factors, we anticipate that tobacco- and alcohol-related content is common in MVs in Vietnam. However, to date, no studies have examined the presence of such content in MVs. Therefore, our study aims to investigate the prevalence of such content in top Vietnamese MVs on YouTube over 9 years (2013 to 2021). Additionally, this study will describe other features related to the depiction of tobacco and alcohol in these MVs.

## Methods

This study followed the guidelines for content analysis studies laid out by Neuendorf (2017) [27].

### Study Sample

The study sample consisted of the top 40 or 50 most viewed Vietnamese MVs released each year between 2013 and 2021 (since 2017, the top 50 MVs were listed instead of the top 40, and 2021 was the most recently available full-year data at the time of the manuscript submission in 2023) [28]. These selections were determined based on viewership data derived from the domestic music chart available on YouTube. A total of 410 MVs were included in this study. The list of most viewed MVs from 2013 to 2017 was last updated in December 2021, while the list of those from 2018 to 2021 was last updated in January 2022.

Among the 410 MVs, 8 original MVs were removed from YouTube for unknown reasons. Because we focused on the MV content, we replaced these missing MVs with the same ones uploaded in later years. When a song had 2 distinct MVs, both were included as separate entries in our analysis since the MV content differed and each was in the top 40/50 as a unique entry. Furthermore, for 3 songs with the same MV listed in the top chart in different years (ie, were uploaded in different years by 2 different accounts), we retained all these MVs instead of eliminating the duplicates. This decision was driven by our objective to investigate the proportion of top MVs in Vietnam featuring tobacco and alcohol content over the years. This approach also acknowledges that audiences have the option to listen to and watch MVs, even if they are repeated from year to year.

### Coding Scheme

#### Overview

We developed a codebook that contained the following sections: general information, lyrics, MV Tobacco content, and MV Alcohol content. The codebook for this study is shown in Multimedia Appendix 1.

#### Section 1: General Information

In this section, general information about the MVs was recorded. Each video was assigned a unique video ID number, and the coding date was recorded. The Vietnamese song’s name, release date, and ranking within its chart year were noted. Age restrictions, indicating who could access the MV, were categorized into no restriction, ≥18 years old, and ≥21 years old. The music genres (ie, mutually exclusive options, including pop, hip-hop/rap, R&B, electronic dance music, and other) and type of MV (ie, not mutually exclusive options, narrative-based MV, lyric, performance/dance, animated MV, and others) were noted. Finally, the presence of tobacco or alcohol references in the song name was recorded as yes or no.

#### Section 2: Lyrics

The song’s lyrics were examined to indicate the presence (1) or absence (0) of mentions of tobacco or alcohol content in the lyrics.

#### Section 3: MV Tobacco Contents

This section coded the depiction of tobacco products in MVs. We coded for specific types of tobacco products shown (cigarettes, e-cigarettes, etc) and their brand/logo, whether tobacco use was shown (eg, lighting a cigarette, person smoking or vaping, etc). Among MVs containing smoking or vaping scenes, the number (ie, 1, 2, 3, or ≥ 4 persons), age (ie, young adult ages 18 to 29 years or adult over the age of 30 years), gender (ie, female, male, or both sexes), and roles of individuals smoking or vaping (ie, singers or others) were categorized. The portrayal of tobacco-related images was assessed, and the cumulative duration of these scenes was recorded (<10 or ≥10 seconds). The presence of warnings about tobacco product harms (yes or no) in MVs was noted.

#### Section 4: MV Alcohol Contents

Similar to the tobacco section, this section coded the depiction of alcohol contents in MVs. We coded images of alcohol containers (ie, beer bottles, cans, etc) and their brand/logos, images of glasses/cups, and scenes of drinking. The presence of scenes showing drunkenness (ie, a person appears to be inebriated) (yes or no) was recorded. Among MVs having drinking scenes, details about the number (ie, 1, 2, 3, or ≥ 4 persons), age (ie, young adult ages 18-29 or adult over the age of 30), gender (ie, female, male, or both sexes), and roles of individuals drinking (ie, singers or others) were documented. The portrayal of alcohol-related images was described, along with the cumulative duration of these scenes (<10 or ≥10 seconds). Next, the presence of warnings about the legal drinking age was assessed.

### MV Coding

The coding team included 4 native Vietnamese speakers with backgrounds in biostatistics or public health. These coders were staff members working at the Hanoi University of Public Health, the University of Medicine and Pharmacy at Ho Chi Minh City, and Pham Ngoc Thach University of Medicine in Vietnam. Before the coding process, a comprehensive 2-hour training session was conducted for the coders, which included 5 parts: (1) introduction to this study (explaining the background and objectives), (2) coding scheme and explaining each variable, (3) working on coding examples from 3 MVs, (4) individual coding practices of 2 MVs, and (5) reconciliation of differences and summary. Each session involved an active discussion. Following the training, the intercoder reliability was established following Neuendorf’s recommendations [29]. Specifically, 10% (41/410) of the overall sample was independently coded by 2 coders each, using a rotating system among 4 coders (A, B, C, and D). The reliability test was performed as follows. First, the 41 videos were coded by pairs of coders, with each coder responsible for 20 or 21 MVs (ie, Coder A: videos 1-20; Coder B: videos 11-30; Coder C: videos 21-41; and Coder D: videos 31-41 and 1-10). This design ensured that each video was coded by 2 different coders, creating overlapping segments for reliability assessment. Then, we calculated the Randolph free-marginal kappa for each variable [30]. The intercoder reliability met the high agreement criterion with α ranging from 0.77 to 1. Multimedia Appendix 2 shows the α for each variable. All discrepancies between coders on the first 41 MVs were resolved by the first author (TPTT). After establishing reliability, the remaining MVs were distributed equally among the 4 coders for the independent coding process (ie, Coder A: videos 42-133, Coder B: videos 134-225, Coder C: videos 226-317, and Coder D: videos 318-410).

The entire coding scheme was implemented within a REDCap (Research Electronic Data Capture) data entry form, enhancing systematic and robust data acquisition.

### Statistical Analysis

Intercoder reliability among the independent coders was assessed using the Randolph free-marginal kappa [30], calculated using the Online Kappa Calculator [31]. A descriptive analysis was conducted to examine the prevalence of tobacco- and alcohol-related MVs across different years and the frequency of associated features. A descriptive analysis of tobacco and alcohol portrayals was also performed. χ² tests were used to assess statistically significant differences in characteristics, including release year, age restriction, and music genres between MVs with and without tobacco (or alcohol) content. The Fisher exact test was used instead of the χ² test when more than 20% of cells had expected frequencies <5. Simple logistic regression models were used to identify the factors associated with tobacco and alcohol contents in MVs. The predictor variables included release year, ranking, age restrictions, music genres, and the type of MV. All statistical analyses were performed with STATA software (version 17.0; StataCorp), and values of *P*<.05 were considered statistically significant.

### Ethical Considerations

This study was reviewed by the Georgia State University Institutional Review Board (H24050) and was determined to be not human subjects research. No personally identifiable information has been collected or analyzed as part of this research.

## Results

Table 1 shows the characteristics of the MVs. Most of the MVs analyzed had no age restriction to access them. In terms of music genres, pop music was the dominant category, accounting for 63.9% (n=262), followed by other types (ie, pop-rap) at 23.4% (n=96). Regarding the type of MVs, a significant proportion were narrative-based (n=281, 68.5%) and performance or dance-oriented (n=287, 70%). The average duration of these MVs was 312 seconds (ie, 5 minutes and 12 seconds).

Of the 410 MVs examined, 36 (8.8%) contained tobacco-related content and 136 (33.2%) featured alcohol-related content. Additionally, 28 (6.8%) out of 410 MVs included both tobacco and alcohol content. Only 2 (5.6%) of 36 MVs with tobacco content and 3 (2.2%) of 136 with alcohol content had age restrictions for audiences aged 18 or older. Notably, hip-hop or rap songs contained more tobacco- and alcohol-related content compared to other music genres. Among narrative-based MVs, nearly half of them contained alcohol-related content. Only 3 (0.7%) and 2 (0.5%) of the MVs mentioned tobacco and alcohol content in the song titles, respectively.

**Table 1:** Characteristics of top Vietnamese YouTube music videos (MVs) between 2013 and 2021 (N=410).

Characteristics		Total, n (%)	MVs containing tobacco-related content			MVs containing alcohol-related content		
			Yes, n (%)	No, n (%)	*P* value^a^	Yes, n (%)	No, n (%)	*P* value
**Total**		410 (100)	36 (8.8)	374 (91.2)	—^b^	136 (33.2)	274 (66.8)	—
**Release year**					.14			.002^c^
	2013	40 (9.8)	3 (7.5)	37 (92.5)		11 (27.5)	29 (72.5)	
	2014	40 (9.8)	0 (0)	40 (100)		11 (27.5)	29 (72.5)	
	2015	40 (9.8)	3 (7.5)	37 (92.5)		15 (37.5)	25 (62.5)	
	2016	40 (9.8)	5 (12.5)	35 (87.5)		7 (17.5)	33 (82.5)	
	2017	50 (12.2)	3 (6)	47 (94)		14 (28)	36 (72)	
	2018	50 (12.2)	6 (12)	44 (88)		23 (46)	27 (54)	
	2019	50 (12.2)	8 (16)	42 (84)		26 (52)	24 (48)	
	2020	50 (12.2)	6 (12)	44 (88)		20 (40)	30 (60)	
	2021	50 (12.2)	2 (4)	48 (96)		9 (18)	41 (82)	
**Age restriction^d^**					.04			.11
	No restriction	406 (99)	34 (8.4)	372 (91.6)		133 (32.8)	273 (67.2)	
	≥18 years old	4 (1)	2 (50)	2 (50)		3 (75)	1 (25)	
	≥21 years old	0 (0)	0 (0)	0 (0)		0 (0)	0 (0)	
**Music genre^d^**					.01			.51
	Rock	0 (0)	0 (0)	0 (0)		0 (0)	0 (0)	
	Pop	262 (63.9)	18 (6.9)	244 (93.1)		88 (33.6)	174 (66.4)	
	Hip-hop/rap	20 (4.9)	6 (30)	14 (70)		7 (35)	13 (65)	
	R&B	6 (1.5)	0 (0)	6 (100)		1 (16.7)	5 (83.3)	
	Electronic dance music	26 (6.3)	0 (0)	26 (100)		5 (19.2)	21 (80.8)	
	Other	96 (23.4)	12 (12.5)	84 (87.5)		35 (36.5)	61 (63.5)	
**Type of MV**								
	Narrative-based MV	281 (68.5)	33 (11.7)	248 (88.3)	.001	131 (46.6)	150 (53.4)	<.001
	Lyric	68 (16.6)	2 (2.9)	66 (97.1)	.06	2 (2.9)	66 (97.1)	<.001
	Performance/dance	287 (70)	29 (10.1)	258 (89.9)	.18	110 (38.3)	177 (61.7)	.001
	Animated	13 (3.2)	0 (0)	13 (100)	.62	1 (7.7)	12 (92.3)	.07
	Other (fan-made and background image)	17 (4.1)	0 (0)	17 (100)	.38	1 (5.9)	16 (94.1)	.02
MV length, mean (SD)		312.1 (101.9)	319 (122.7)	311.5 (99.9)		336.1 (117.7)	300.2 (91)	

^a^*P* values were calculated by the Fisher exact test (except for *P*=.002) to assess statistically significant differences in characteristics between MVs with and without tobacco (or alcohol) content.

^b^Not applicable.

^c^χ^2^ test was used instead.

^d^Mutually exclusive response.

Table 2 presents the predictors of tobacco and alcohol contents in MVs. Compared to 2013, MVs released in 2019 had an increased likelihood of containing alcohol content. MVs with a higher ranking (ie, a lower number means a higher rank) were more likely to contain alcohol-related content. The hip-hop/rap genre was more likely to contain tobacco content (odds ratio [OR]=5.81, 95% CI=1.99-16.93) compared to pop. Regarding types of MVs, narrative-based MVs had higher odds of having both tobacco content (OR=5.59, 95% CI=1.68-18.58) and alcohol content (OR 21.66, 95% CI=8.59-54.58) compared to nonnarrative-based MVs. Additionally, performance/dance MVs had an increased likelihood of including alcohol content (OR 2.32, 95% CI=1.41-3.80) compared to MVs that were not focused on performance/dance.

**Table 2:** The predictors of tobacco and alcohol content in 410 Vietnamese YouTube music videos (MVs).

Characteristics			MVs containing tobacco-related content, OR^a^ (95% CI)		MVs containing alcohol-related content, OR (95% CI)
**Release year (versus 2013)**					
	2014	N/A^b^		1 (0.37-2.67)	
	2015	1 (0.19-5.28)		1.58 (0.62-4.07)	
	2016	1.76 (0.39-7.93)		0.56 (0.19-1.63)	
	2017	0.79 (0.15-4.13)		1.03 (0.40-2.60)	
	2018	1.68 (0.39-7.19)		2.25 (0.92-5.47)	
	2019	2.35 (0.58-9.51)		2.86* (1.17-6.94)	
	2020	1.68 (0.39-7.19)		1.76 (0.72-4.30)	
	2021	0.51 (0.08-3.24)		0.58 (0.21-1.57)	
Ranking in the top chart			1.01 (0.99-1.04)		0.98* (0.97-1)
Age restriction (≥18 years old versus no restriction)			10.94* (1.49-80.13)		6.16 (0.63-59.76)
**Music genres (versus pop)**					
	Hip-hop/rap	5.81** (1.99-16.93)		1.06 (0.41-2.76)	
	R&B	N/A		0.40 (0.05-3.44)	
	Electronic dance music	N/A		0.47 (0.17-1.29)	
	Other	1.94 (0.90-4.19)		1.13 (0.70-1.85)	
**Type of MVs**					
	Narrative-based MV (yes versus no)	5.59** (1.68-18.58)		21.66*** (8.59-54.58)	
	Lyric (yes versus no)	0.27 (0.06-1.17)		0.05*** (0.01-0.20)	
	Performance/dance (yes versus no)	1.86 (0.79-4.38)		2.32*** (1.41-3.80)	
	Animated MV (yes versus no)	N/A		0.16 (0.02-1.26)	
	Other (yes versus no)	N/A		0.12* (0.02-0.91)	

^a^OR: odds ratio.

^b^N/A: not applicable.

*P<.05, ** *P*<.01, *** *P*<.001.

Multimedia Appendix 3 illustrates the proportion of tobacco- and alcohol-related content in the 410 MVs analyzed. While 7.6% (n=31) of MVs depicted the actual use of tobacco products, 3.7% (n=15) contained just images of tobacco products (eg, a cigarette pack lying on a table). In terms of alcohol-related content, 24.9% (n=102) of MVs featured images of alcohol containers, such as wine or beer bottles and cans. Additionally, 23.4% (n=96) included images of wine or beer glasses or cups, while 21.2% (n=87) depicted scenes of individuals consuming alcohol. Furthermore, 8% (n=33) of MVs portrayed someone in a state of inebriation.

**Figure 1:**
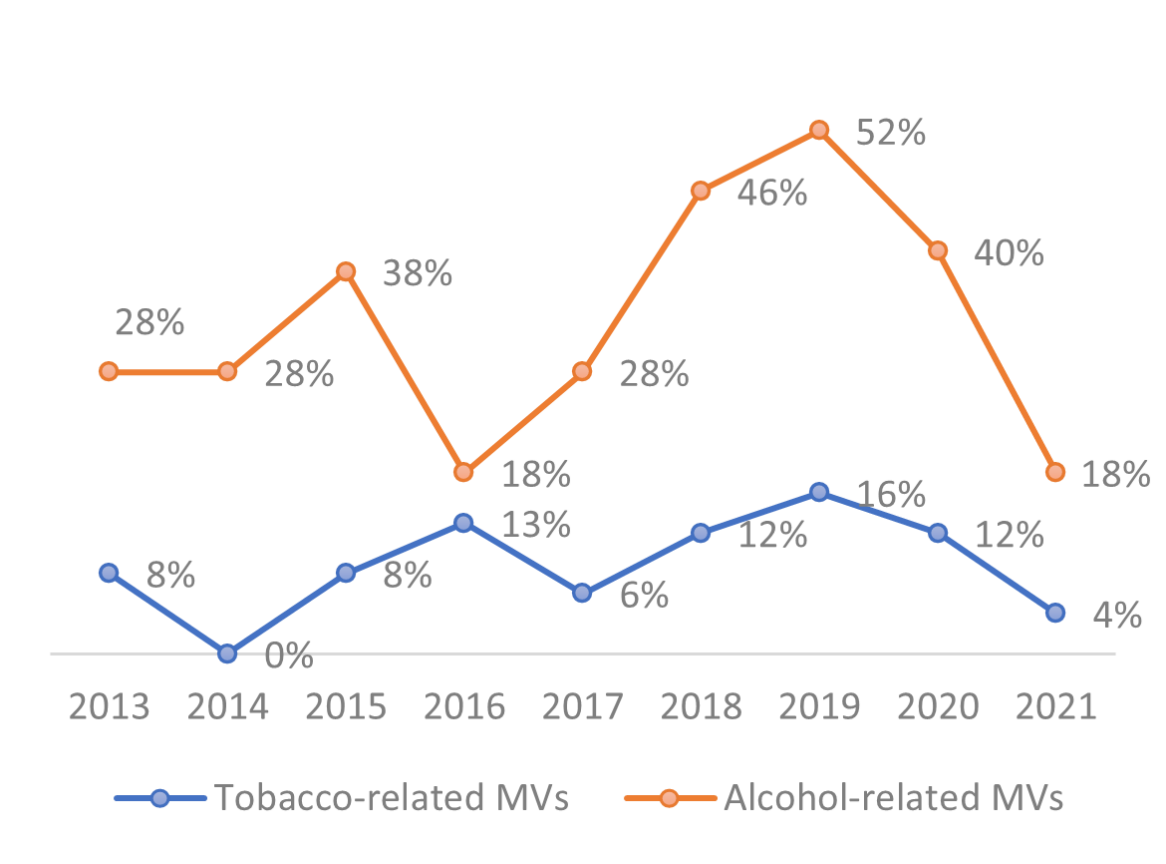
The prevalence of music videos (MVs) containing tobacco and alcohol-related content across the years.

The prevalence of MVs containing tobacco- and alcohol-related content had a fluctuating pattern over the years, as illustrated in Figure 1. Notably, for alcohol, the proportion of videos with alcohol content increased every year between 2016 and 2019, when more than half of the MVs contained alcohol-related imagery.

Table 3 describes the portrayal of tobacco in MVs. The following numbers depict the prevalence of MVs containing tobacco-related content (n=36, 8.8%). Cigarettes were the most frequently shown product (n=28, 77.8%), followed by bamboo waterpipe tobacco (n=3, 8.3%). Only 2.8% (n=1) of the MVs displayed tobacco brands, and 5.6% (n=2) included health warnings about tobacco harm. Tobacco imagery was commonly seen at parties, bars, and pubs (n=13, 36.1%), as well as during dancing and singing scenes (n=12, 33.3%). In 19.4% (n=7) of songs, tobacco content was mentioned in the lyrics. Regarding actual use of tobacco products, tobacco use was typically depicted with one person, often a young adult male, and 38.7% (n=12) showed singer(s) smoking. Only 5.6% (n=2) of MVs containing tobacco content had warnings about the harm of tobacco use.

**Table 3:** Portrayal of tobacco in 36 Vietnamese YouTube music videos (MVs) with tobacco content.

Characteristics			n (%)
**Tobacco products**			
	Cigarette	28 (77.8)	
	Bamboo waterpipe tobacco	3 (8.3)	
	Hookah	1 (2.8)	
	Cigarillos/little cigar	1 (2.8)	
	Large cigar	3 (8.3)	
	Other (pipes, roll-your-own cigarettes)	2 (5.6)	
**Images of tobacco brand/logo^a^**			
	No	35 (97.2)	
	Yes	1 (2.8)	
**Tobacco portrayals**			
	Shown in sad scenes depicting someone crying, dying, etc	8 (22.2)	
	Shown when someone was stressed or anxious	7 (19.4)	
	Shown during dancing or singing scenes	12 (33.3)	
	Shown at parties, bars, or pubs	13 (36.1)	
**Duration of tobacco contents^a^**			
	<10 seconds	19 (52.8)	
	≥10 seconds	17 (47.2)	
**Warning or disclaimer about tobacco harmfulness^a^**			
	No	34 (94.4)	
	Yes	2 (5.6)	
**Tobacco mentioned in the lyrics of the song^a^**			
	No	29 (80.6)	
	Yes	7 (19.4)	
**Number of smoker(s)^a,b^**			
	1	18 (58.1)	
	2	7 (22.6)	
	3	2 (6.5)	
	≥4	4 (12.9)	
**Age of smoker(s)^b^**			
	Young adult (18 to 29 years)	24 (77.4)	
	Adult (30+ years)	10 (32.3)	
**Person(s) smoking^a^**			
	Singer(s)	12 (38.7)	
	Dancer or featured star (not singer)	23 (74.2)	
	Could not be determined	1 (3.2)	
**Biological sex of smoker(s)^a,b^**			
	Female	2 (6.5)	
	Male	26 (83.9)	
	Both sexes	3 (9.7)	

^a^Mutually exclusive response.

^b^Among MVs containing actual tobacco use scenes (n=31).

Table 4 presents the portrayal of alcohol in the MVs. Images of alcoholic beverage brands or logos appeared in 26 (19.1%) MVs. There was a high proportion of MVs showing alcohol images in parties, bars, or pubs (n=96, 70.6%). Alcohol-related content was mentioned in lyrics in 45 (33.1%) MVs. Only 1.5% (n=2) of MVs included warnings about drinking being restricted to individuals 18 years old and above. In MVs depicting drinking, it frequently involved groups (ie, more than 4 people) (n=45, 51.7%), among young adults of both sexes (n=53, 60.9%), and 56 MVs (64%) showed singers drinking. Only 2 MVs (1.5%) containing alcohol content had warning about drinking being restricted to individuals 18 years old and above.

**Table 4:** Portrayal of alcohol in 136 Vietnamese YouTube music videos (MVs) with alcohol content.

Characteristics		n (%)
**Images of wine/beer brand/logo^a^**		
	No	110 (80.9)
	Yes	26 (19.1)
**Alcohol portrayals**		
	Shown in sad scenes depicting someone crying, dying, etc	23 (16.9)
	Shown when someone is stressed or anxious	14 (10.3)
	Shown during dancing or singing scenes	32 (23.5)
	Shown at parties, bars, or pubs	96 (70.6)
**Duration of alcohol content^a^**		
	<10 seconds	28 (20.6)
	≥10 seconds	108 (79.4)
**Warning about drinking being restricted to individuals aged 18 years and above^a^**		
	No	134 (98.5)
	Yes	2 (1.5)
**Alcohol mentioned in the lyrics of the song^a^**		
	No	91 (66.9)
	Yes	45 (33.1)
**Number of drinker(s)^a,b^**		
	1	23 (26.4)
	2	14 (16.1)
	3	5 (5.7)
	≥4	45 (51.7)
**Age of drinker(s)^b^**		
	Young adult	82 (94.3)
	Adult	21 (24.1)
**Who the drinker(s) are^b^**		
	Singer(s)	56 (64.4)
	Dancer or featured star (not singer)	75 (86.2)
**Biological sex of the drinker(s)^a,b^**		
	Female	6 (6.9)
	Male	28 (32.2)
	Both sexes	53 (60.9)

^a^Mutually exclusive response.

^b^Among MVs containing drinking scenes (n=87).

## Discussion

### Principal Results

Tobacco and alcohol content were prevalent in top Vietnamese YouTube MVs. Our findings indicate that 8.8% (n=36) of MVs from 2013 to 2021 contained tobacco content, and 33.2% (n=136) featured alcohol content. This aligns with a study by Gruber et al [32], where alcohol was present in 34.5% of MVs and tobacco products were present in 10% of MVs broadcast on television channels in the United States in 2001. However, these rates were lower than recent studies in the United Kingdom [33], which showed alcohol and tobacco imagery in 45% and 22% of YouTube MVs, respectively, and in the United States, where nearly one-fourth of popular MVs from 2018 to 2020 contained tobacco imagery [34]. These differences across countries could be attributed to cultural attitudes toward tobacco use, regulatory frameworks, and social norms. The higher prevalence of alcohol content compared to tobacco content in the MVs could be attributed to widespread direct endorsement by the artists who have financial associations with alcoholic products [33]. Additionally, alcohol is often portrayed as a social lifestyle and is more widely accepted in social settings such as parties, celebrations, and nightlife, which are common themes in MVs.

We observed an increase in the proportion of MVs containing tobacco and alcohol from 2013 to 2019, followed by a decrease in 2020 and 2021. Several factors might contribute to this increase, including evolving cultural norms, shifts in marketing strategies by the tobacco and alcohol industries, and changes in popular music trends. Conversely, the subsequent decline could be attributed to the impact of the COVID-19 pandemic, as suggested in a previous study [34]. For instance, the parody song “Ghen Cô Vy,” which emphasized handwashing and personal hygiene to prevent COVID-19, went viral and topped the charts in 2020. Another factor could be the variation in the number of narrative-based MVs, being highest in 2019 and lowest in 2020 and 2021. Further monitoring is necessary to validate these trends.

### Comparison With Prior Works

Our findings revealed that the hip-hop genre was associated with a higher rate of tobacco content in Vietnamese YouTube MVs. This was consistent with previous studies indicating that nearly half of tobacco-related incidences appeared in hip-hop songs among the Billboard top tracks in the United States from 2013 to 2017 [35]. Hip-hop artists often serve as role models for their fans owing to their prominence and the social, cultural, and political commentary in their music, which includes tobacco use [35]. While alcohol is likely to appear across various music genres, it is more common in hip-hop and pop MVs [22,32,33], consistent with our study.

The prevalence of smoking and drinking in society was reflected in the portrayals of tobacco and alcohol in MVs. Cigarettes were the most commonly depicted tobacco product in MVs, followed by bamboo waterpipe tobacco—a traditional tobacco product—representing the most commonly used types of tobacco in Vietnam (36.7% of men used cigarettes, and 13.7% used bamboo waterpipes) [25]. We did not find any MVs containing new tobacco products such as e-cigarettes and heated tobacco products, which are not popular in Vietnam, especially among adults, with a prevalence of less than 1% [25]. There is a notable disparity in smoking prevalence in society between men (45.3%) and women (1.1%) [25], as reflected in the MVs, where scenes of male smokers were very common, accounting for 83.9% (n=26) of all the MVs compared to female smokers at 6.5% (n=2). In terms of drinking behavior, there was also a disparity in alcohol consumption between men and women, with approximately 60% of men reporting past-week alcohol consumption compared to only 5% of women [36]. However, the open social norms surrounding women’s participation in the modern “drinking culture” [37] could contribute to a high proportion of MVs depicting both male and female characters drinking (n=53, 60.9%). Additionally, smoking appeared to be solitary behavior, with more than half of the MVs depicting a scene with only 1 person smoking. In contrast, drinking was portrayed as a social behavior in Vietnam, with 51.7% (n=45) of the MVs showing a group of people (ie, 4 or more persons) drinking together.

One-third of MVs with tobacco content showcased singers engaging in smoking, while two-thirds of videos with alcohol content featured singers consuming alcohol. These depictions likely contribute to the formation of positive attitudes toward tobacco and alcohol use. It is noteworthy that the primary audience for these artists comprises adolescents and young adults, as they make up the predominant demographic using social media platforms like YouTube to follow music and swiftly adopt new social trends. Moreover, considering that a substantial proportion of individuals depicted as smokers or drinkers in MVs were young adults (n=24, 77.4% for tobacco and n=82, 94.3% for alcohol) in our study, the societal impact is considerable. Given that many of these singers boast millions of fans, including children, the potential influence on society is profound, effectively turning them into spokespeople for the industry [17]. Adolescents and young adults frequently look up to their idols, often seeking to emulate various aspects of their behavior. Consequently, the portrayal of tobacco and alcohol in MVs poses a significant public health risk to these age groups, potentially diminishing their awareness of the risks associated with tobacco and alcohol use, heightening their susceptibility to use tobacco, and even leading to initiation [4,22,38].

### Implications for Regulations

Vietnam’s regulations on tobacco and alcohol content in online platforms reveal a notable gap. Circular No 25/2018/TT-BVHTTDL, issued by the Ministry of Culture Sports and Tourism, restricts the depiction of actors using tobacco in theater and cinematic works. Additionally, Decree No 24/2020/ND-CP outlines regulations related to certain provisions of the law on preventing and combating the harms of alcoholic beverages. These regulations require approval from the authorized agency based on recommendations of the Evaluation Council for scenes depicting tobacco use or alcohol consumption in artistic works. The use of such images is permissible only when necessary for portraying historical characters, recreating a specific historical period, or critiquing and condemning smoking or drinking. Films or movies must be classified according to appropriate age groups and include health warnings regarding the harmful effects of tobacco and alcohol, such as “drinking alcohol may cause traffic accidents” and “persons under 18 years of age are forbidden to drink alcohol” either in text or images. However, it is unclear whether these regulations would apply to MVs, which are digital videos and are not released in the form of a tape or film. Our study found that most of the tobacco and alcohol content depicted in top Vietnamese MVs was not accompanied by any health warnings.

Managing activities on the internet, including MVs on platforms like YouTube, is challenging, as Vietnam is grappling with issues related to advertising and sponsorship on the internet. The widespread use of sophisticated online techniques, including social media, for promoting tobacco and alcohol products has led to challenges in regulation [16,39]. Artists, whether intentionally or inadvertently, become effective and powerful marketing tools for these products by incorporating images of tobacco and alcohol in their MVs on social media platforms like YouTube [6,33,34]. Moreover, in Vietnam, certain exceptions exist for drinking, notably in the case of highlanders and specific ethnic groups. For instance, the traditional straw liquor of highlanders and other ethnic communities is regarded as a cultural expression cultivated over generations. Consequently, these images may provoke controversial opinions and pose challenges in adherence to regulations.

Although no laws specifically impose age restrictions for MVs, the Tobacco Control Law No 09/2012/QH13, issued by the National Assembly in 2012, prohibits the depiction of children under 18 years old smoking. Regulations related to alcohol use among children under 18 years were issued more recently. This might explain our findings, where the presence of tobacco content was significantly associated with MVs having an age restriction, while this was not seen for alcohol content. However, it is important to emphasize that the number of MVs containing tobacco and alcohol content with age restrictions remained very low.

YouTube does not allow content showing “harmful or dangerous acts involving minors,” such as minors drinking alcohol or using tobacco [40]. While YouTube’s Community Guidelines are universal policies that apply to users worldwide, it also has procedures to respond appropriately to valid legal requests that are based on relevant local laws [41]. For example, YouTube permits branding and informational advertising for alcoholic beverages in 68 countries, with the exception of 4. Vietnam only allows beverages with less than 5.5% alcohol by volume, Ecuador with less than 5%, and India and Indonesia with 0% alcohol [42]. Tobacco advertising on YouTube is prohibited. In addition, videos with tobacco content fall under the “limited or no ads” monetization state, meaning that content creators will not be able to receive revenues for advertisements placed in their videos. However, this information is not shown to the viewers and might not be applicable to MVs, as “artistic content such as MVs may contain elements such as inappropriate language, references to soft drug use, or nonexplicit sexual themes, and still be suitable for advertising” [43]. Content that depicts tobacco might be age-gated or restricted on YouTube. However, as our results showed, only 5.6% (n=2) of MVs with tobacco content had age restrictions. Thus, young people are not sufficiently protected from alcohol- and tobacco-related content in MVs on YouTube. Considering the billions of views that alcohol and tobacco content receive on YouTube and their potentially harmful effects, there is a pressing need to regulate such content. Given the other restrictions YouTube has put in place [44], it should expand these rules to include any portrayals of tobacco (and possibly alcohol) regardless of the type of video—at least for monetization purposes if an outright ban is not feasible. Governments should consider implementing the Framework Convention on Tobacco Control requirements on the internet to reduce protobacco content. Our study also revealed novel findings, showing that mentions of tobacco or alcohol in song lyrics were prevalent. This emphasizes the importance of regulating not only visual imagery but also lyrical content.

### Limitations

This study has several limitations. First, some songs that are popular among adolescents and young adults were not included in this study because they did not make it onto the top charts. Second, although this study included only top MVs on YouTube, MVs can also be posted and viewed via other popular websites such as Spotify, Zing MP3, and social media platforms like Facebook. Third, this study was unable to identify the characteristics of individuals who viewed each video and their exposure to tobacco and alcohol content in the MVs. Therefore, further studies are needed to investigate how exposure to MVs containing tobacco and alcohol content impacts perceptions and behaviors related to smoking and drinking. Finally, the small sample size of tobacco-related MVs likely did not provide sufficient power to detect significant effects of the release year on tobacco content.

### Conclusions

In conclusion, our analysis of top Vietnamese MVs from 2013 to 2021 revealed a concerning prevalence of tobacco and alcohol content. Approximately one-third of the MVs contained alcohol content, while nearly 1 in 10 featured tobacco content. Additionally, most of the tobacco and alcohol content was depicted in MVs without any warnings or health disclaimers. This study highlighted a regulatory gap in addressing such content online, emphasizing the need for more stringent regulations and age restrictions on platforms like YouTube. Furthermore, there is a critical need for additional research to understand how exposure to MVs influences the perceptions and behaviors of adolescents and young adults regarding smoking and drinking.

## Appendix

Multimedia Appendix 1 Updated codebook.

Multimedia Appendix 2 The α for each variable.

Multimedia Appendix 3 The proportion of tobacco- and alcohol-related content in the music videos (N=410).

## Abbreviations

APS: advertising, promotion, and sponsorship
MV: music video
OR: odds ratio
REDCap: Research Electronic Data Capture

## Data Availability

The data sets generated during and/or analyzed during this study are available from the corresponding author upon reasonable request.
