# Supplementary material for: Tobacco and Alcohol Content in Top Vietnamese YouTube Music Videos: Content Analysis
Source: J Med Internet Res. 2024 Nov 8;26:e55555. doi: 10.2196/55555 (PMC11584539; doi:10.2196/55555)
Supplement: Multimedia Appendix 1 [file jmir_v26i1e55555_app1.docx]

**RESEARCH PROTOCOL FOR TOBACCO AND ALCOHOL**

**IN YOUTUBE MUSIC VIDEOS CONTENT ANALYSIS**

**I. Summary of variables coded by coders**

| **No.** | **Variables names** | **Notation** | **Variable type** |
| --- | --- | --- | --- |
| **Section 1. General information** | | | |
| 1.1 | Unique ID | id | Numeric |
| 1.2 | Date of access | date | Date |
| 1.3 | Song name | name | Text |
| 1.4 | Release date | release_date | Date |
| 1.5 | Ranking song of the year | rank | Numeric |
| 1.6 | Music video (MV) length | length | Numeric |
| 1.7 | No. of views | views | Numeric |
| 1.8 | No. of likes | likes | Numeric |
| 1.9 | No. of dislikes | dislikes | Numeric |
| 1.10 | Age restriction | age_mv | Categorical |
| 1.11 | Tobacco mentioned in the name of the song | name_tob | Binary (Y/N) |
| 1.12 | Alcohol mentioned in the name of the song | name_alc | Binary (Y/N) |
| 1.13 | Type of music | type_music | Categorical |
|  |  | type_music_other | Text |
| 1.14 | Type of MV | type_mv_narative | Binary (Y/N) |
|  |  | type_mv_lyric | Binary (Y/N) |
|  |  | type_mv_performance | Binary (Y/N) |
|  |  | type_mv_animated | Binary (Y/N) |
|  |  | type_mv_other | Text |
| **Section 2. Lyrics** | | | |
| 2.1 | Tobacco mentioned in the lyrics | lyric_tob | Binary (Y/N) |
| 2.2 | Alcohol mentioned in the lyrics | lyric_alc | Binary (Y/N) |
| **Section 3. MV – Tobacco contents** | | | |
| 3.1 | Images of tobacco products (not used) | tob_image | Binary (Y/N) |
| 3.2 | Images of specific tobacco types | tob_cig | Binary (Y/N) |
|  |  | tob_wp | Binary (Y/N) |
|  |  | tob_ecig | Binary (Y/N) |
|  |  | tob_hookah | Binary (Y/N) |
|  |  | tob_littlecigar | Binary (Y/N) |
|  |  | tob_htp | Binary (Y/N) |
|  |  | tob_other | Text |
| 3.3 | Images of using tobacco (lighting a cigarette/waterpipe tobacco; smoking, vaping; constructing an e-cigarette) | tob_smk | Binary (Y/N) |
| 3.4 | Images of tobacco brand/logo | tob_logo | Binary (Y/N) |
|  |  | tob_brandname | Text |
| 3.5 | Number of smoker(s) and vaper(s) | tob_person_num | Scale |
| 3.6 | Age of smoker/vaper | tob_person_age_x1 | Binary (Y/N) |
|  |  | tob_person_age_x2 | Binary (Y/N) |
|  |  | tob_person_age_x3 | Binary (Y/N) |
|  |  | tob_person_age_x77 | Binary (Y/N) |
| 3.7 | Smoker(s) or vaper(s) is a singer? | tob_person_singer | Categorical |
| 3.8 | Biological sex of smoker(s) or vaper(s) | tob_person_sex | Categorical |
| 3.9 | Tobacco portrayals | tob_portrayal_x1 | Binary (Y/N) |
|  |  | tob_portrayal_x2 | Binary (Y/N) |
|  |  | tob_portrayal_x3 | Binary (Y/N) |
|  |  | tob_portrayal_x4 | Binary (Y/N) |
|  |  | tob_portrayal_text | Text |
| 3.10 | Duration of tobacco contents | tob_long | Categorical |
| 3.11 | Warning contents about tobacco/ENDs harmfulness | tob_warn | Binary (Y/N) |
| **Section 4. MV – Alcohol contents** | | | |
| 4.1 | Images of wine bottles/beer bottles (not drinking) | alc_image_bottle | Binary (Y/N) |
| 4.2 | Images of wine/beer glasses (not drinking) | alc_image_glass | Binary (Y/N) |
| 4.3 | Images of drinking | alc_drinking | Binary (Y/N) |
| 4.4 | Images of someone who gets drunk | alc_drunk | Binary (Y/N) |
| 4.5 | Images of wine/beer brand/logo | alc_logo | Binary (Y/N) |
|  |  | alc_brandname | Text |
| 4.6 | Number of people drinking | alc_person_num | Scale |
| 4.7 | Age of persons drinking | alc_person_age_x1 | Binary (Y/N) |
|  |  | alc_person_age_x2 | Binary (Y/N) |
|  |  | alc_person_age_x3 | Binary (Y/N) |
|  |  | alc_person_age_x77 | Binary (Y/N) |
| 4.8 | Drinker(s) is singer | alc_person_singer | Categorical |
| 4.9 | Biological sex of drinker(s) | alc_person_sex | Categorical |
| 4.10 | Alcohol portrayals | alc_portrayal_x1 | Binary (Y/N) |
|  |  | alc_portrayal_x2 | Binary (Y/N) |
|  |  | alc_portrayal_x3 | Binary (Y/N) |
|  |  | alc_portrayal_x4 | Binary (Y/N) |
|  |  | alc_portrayal_text | Text |
| 4.11 | Duration of alcohol contents | alc_long | Categorical |
| 4.12 | Warning contents regarding drinking | alc_warn | Binary (Y/N) |

**II. Variables descriptions**

**Section 1. General information**

- 1. Unique ID (id): Video ID number
  2. Access date: Date when coder conducts the coding (DD/MM/YY)
  3. Song name (name): Name of the song in Vietnamese
  4. Release date (release_date): The date when the song was released

*[Ranging from 2013 to 2021]*

- 1. Ranking song of the year (rank): The ranking number of that song in the release year

*[From 2013-2016: ranging from 1 to 40]*

*[From 2017-2021: ranging from 1 to 50]*

- 1. MV length (length): Length of MV in seconds
  2. Number of views (views): Number of views of MV as of the access date
  3. No. of likes (likes): Number of likes at access date.
  4. No. of dislikes (dislikes): Number of dislikes at access date

1.10. Age restriction (age_mv): who can access the MV?

1. No age restriction
2. <18 years old restriction
3. <24 years old restriction

1.11. Tobacco mentioned in the name of the song (name_tob): the name of the song contains any words/contents related to tobacco/vaping. All other drugs or cannabis-related contents are excluded [Y=1/N=0]

1.12. Alcohol mentioned in the name of the song (name_alc): the name of the song contains any words/contents related to alcohol/drinking [Y=1/N=0]

1.13. Type of music (type_music)

1. Rock: it is characterized by the use of complex melodies and rhythms, with fairly fixed instrumentation (basically electric guitar, electric bass, keyboard and drums) and with an energetic rhythm underlined by the power of the bass and the drums, all of which is often linked to an anti-authoritarian and provocative attitude
2. Pop: Popular music characterized by catchy melodies, simple lyrics, and a focus on commercial appeal. Examples include Taylor Swift, Ed Sheeran, and Ariana Grande
3. Hip-hop/Rap: it is characterized by its electronic base and for being associated with alternative manifestations such as break dance or graffiti. It uses a technique named “Rapping” which is poetry spoken rhythmically.
4. RnB: It is characterized by being a mainly vocal genre, typical of solo singers or grouped in quartets or quintets that accompany their voice with the interventions of a choir.
5. EDM: music production made with various electronic devices. For example, synthesizers, digital recorders, software, computers, etc.
6. Other (type_music_other): specify other types of music not mentioned above.

77. Cannot be determined

1.14. Type of MV (type_MV): Types of MVs

- Narrative-based MV (type_MV_1): regular MV is a visual representation or short film that accompanies a song or piece of music. It typically combines music, imagery, and storytelling to enhance the listener's experience and convey the artistic vision of the musician or band [Y=1/N=0]
- Lyric (type_MV_2): a type of video that accompanies a song and displays the song's lyrics in sync with the music. It is primarily designed to help viewers follow along with the lyrics while listening to the song [Y=1/N=0]
- Performance/Dance (type_MV_3): Performances focus on showcasing the artist or band performing the song. It typically features footage of live performances, studio recordings, or staged performances in a visually engaging manner; Dances center around choreography and showcase professional dancers or the artists themselves performing intricate dance routines. [Y=1/N=0]
- Animated music videos (type_MV_4): everything from a simple 2D animation to stop motion or computer-generated imagery. [Y=1/N=0]
- Other (type_MV_5): specify other types of MV not mentioned above. [Y=1/N=0]

**Section 2. Lyrics**

2.1. Tobacco mentioned in the lyrics (lyric_tob): The lyric of the song contains any words/contents related to tobacco/vaping. All other drugs or cannabis-related contents are excluded [Y=1/N=0]

2.2. Alcohol mentioned in the lyrics (lyric_alc): The lyric of the song contains any words/contents related to alcohol/drinking [Y=1/N=0]

**Section 3. MV – Tobacco contents**

3.1. Images of tobacco products(tob_image): The MV contains scenes in which tobacco products/ENDS appear, but they are NOT used. [Y=1/N=0]

Note. If (0) is selected, **skip to 3.3.**

3.2. Images of specific tobacco types: Specify the type of tobacco products/ENDs that appear in MV

(tob_cig) Cigarettes [Y=1/N=0]

(tob_ecig) E-cigarette [Y=1/N=0]

(tob_wp) Bamboo waterpipe tobacco [Y=1/N=0]

(tob_hookah) Hookah [Y=1/N=0]

(tob_littlecigar) Cigarillos/Little cigars [Y=1/N=0]

(tob_largecigar) Large cigars [Y=1/N=0]

(tob_htp) Heated tobacco products [Y=1/N=0]

(tob_other) Specify other tobacco products (pipes, roll-your-own cigarettes,…)

3.3. Images of using tobacco (tob_smk): The MV contains scenes in which person(s) lighting a cigarette/waterpipe tobacco; smoking, vaping; constructing an e-cigarette. It also includes instances where only a part of a body, such as the mouth or face, is shown smoking/vaping, or a hand holding a cigarette/ENDs or constructing ENDs[Y=1/N=0].

Note. If (tob_image) = 0 AND (tob_smk) = 0, **skip to Section 4.**

3.4. Images of tobacco brand/logo (tob_logo): The MV contains scenes in which the logo or brand name of tobacco products/ENDS appears [Y=1/N=0]

(tob_brandname) Specify brand names of tobacco products/ENDS.

3.5. Number of smoker(s) and vaper(s) (tob_person_num): The highest number of smoker(s) and vaper(s) appears in one scene in the MV

*Only asked if (tob_smk)=1*

1. One

2. Two

3. Three

4. ≥ Four

3.6. Age of smoker/vaper: Determine the age of smoker(s) and vaper(s) in the MV, NOT in reality.

*Only asked if (tob_smk)=1*

(tob_person_age_x1) Youth under age 18 (e.g., in school uniform) [Y=1/N=0]

(tob_person_age_x2) Young adults ages 18-29 [Y=1/N=0]

(tob_person_age_x3) Adults over the age of 30 [Y=1/N=0]

(tob_person_age_x77) Cannot be determined [Y=1]

Note. If you cannot determine the age, code as 1 in (tob_person_age_x3) - adults over the age of 30. If the full face or body is not fully shown, and you cannot determine their age, code as 1 in (tob_person_age_x77).

3.7. Smoker(s) or vaper(s) is singer? (tob_person_singer): Who is a smoker or vaper?

*Only asked if (tob_smk)=1*

(tob_person_singer_x1) Singer(s) [Y=1/N=0]

(tob_person_singer_x2) Others such as dancer or starring (not singer) [Y=1/N=0]

(tob_person_singer_x77) Cannot be determined [Y=1]

3.8. Biological sex of smoker(s) or vaper(s) (tob_person_sex): Sex can be judged based on smokers’ or vapers’ physical appearance.

*Only asked if (tob_smk)=1*

1. All smokers/vapers are female

2. All smokers/vapers are male

3. Smokers/vapers are both males and females

77. At least one person’s sex cannot be determined

3.9. Tobacco portrayals: Description of how tobacco-related images are portrayed.

*Only asked if (tob_image) = 1 OR (tob_smk) = 1*

(tob_portrayal_x1): Whether tobacco-related images are shown in sad scenes depicting someone crying, dying, breaking up, etc. [Y=1/N=0]

(tob_portrayal_x2): Whether tobacco-related images are shown when someone is stressed or anxiety [Y=1/N=0]

(tob_portrayal_x3): Whether tobacco-related images are shown during dancing or singing scenes [Y=1/N=0]

(tob_portrayal_x4): Whether tobacco-related images are shown at parties, bars, or pubs [Y=1/N=0]

(tob_portrayal_text): Provide a detailed description of the scenes where tobacco-related content is portrayed.

3.10. Duration of tobacco contents (tob_long): The total duration of all tobacco-related scenes combined in the MV?

*Only asked if (tob_image) = 1 OR (tob_smk) = 1*

1. <10 seconds

2. ≥10 seconds

3.11. Warning contents about tobacco/ENDs harmfulness (tob_warn): The MV contains scenes that warn about the harmfulness of tobacco/ENDs [Y=1/N=0]

**Section 4. MV – Alcohol contents**

4.1. Images of wine/beer bottles/cans (alc_image_bottle): The MV contains scenes in which wine/beer bottles/cans appear, but they are NOT drinking [Y=1/N=0]

4.2 Images of wine/beer glasses/cup (alc_image_glass): The MV contains scenes in which wine/beer glasses/cups appear, but they are NOT drinking [Y=1/N=0]

4.3. Images of drinking alcohol (alc_drinking): The MV contains scenes in which person(s) is drinking. It also includes instances where only a part of a body, such as the mouth or face, is shown drinking or a hand holding a bottle/glass/cup[Y=1/N=0]

4.4. Images of someone who gets drunk (alc_drunk): The MV contains scenes in which person(s) get drunk? [Y=1/N=0]

Note. If (alc_image_bottle)=0 AND (alc_image_glass)=0 AND (alc_drinking)=0, **skip to section 5.**

4.5. Images of wine/beer brand/ logo (alc_logo): The MV contains scenes in which the logo or brand name of alcoholic beverages appears [Y=1/N=0].

(alc_brandname) Specify brand names of alcoholic beverages.

4.6. Number of people drinking (alc_person_num): The highest number of drinking person(s) appear(s) in one scene in the MV

*Only asked if (alc_drinking)=1*

1. One

2. Two

3. Three

4. ≥ Four

4.7. Age of persons drinking: Determine the age of drinking person(s) in the MV, NOT in reality.

*Only asked if (alc_drinking)=1*

(alc_person_age_x1) Youth under age 18 (e.g., in school uniform) [Y=1/N=0]

(alc_person_age_x2) Young adults ages 18-29 [Y=1/N=0]

(alc_person_age_x3) Adults over the age of 30 [Y=1/N=0]

(alc_person_age_x77) Cannot be determined [Y=1]

If the full face or body is not fully shown, and you cannot determine their age, code as 1 in (alc_person_age_x77).

4.8. Drinker(s) is singer? (alc_person_singer): Who is drinker?

*Only asked if (alc_drinking)=1*

(alc _person_singer_x1) Singer(s) [Y=1/N=0]

(alc _person_singer_x2) Others such as dancer or starring (not singer) [Y=1/N=0]

(alc _person_singer_x77) Cannot be determined [Y=1]

4.9. Biological sex of drinker(s) (alc_person_sex): Sex can be judged based on drinkers’ physical appearance.

*Only asked if (alc_drinking)=1*

1. All smokers/vaper are female

2. All smokers/vaper are male

3. Smokers/vapers are both males and females

77. At least one person’s sex cannot be determined

4.10. Alcohol portrayals: Description of how alcohol-related images are shown.

*Only asked if (alc_image_bottle)=1 OR (alc_image_glass)=1 OR (alc_drinking)=1 OR (alc_drunk)=1*

(alc_portrayal_x1): Whether alcohol-related images are shown in sad scenes depicting someone crying, dying, breaking up, etc. [Y=1/N=0]

(alc_portrayal_x2): Whether alcohol-related images are shown when someone is stressed or anxiety [Y=1/N=0]

(alc_portrayal_x3): Whether alcohol-related images are shown during dancing or singing scenes [Y=1/N=0]

(alc_portrayal_x4): Whether alcohol-related images are shown at parties, bars, or pubs [Y=1/N=0]

(alc_portrayal_text): Provide a detailed description of the scenes where alcohol-related content is portrayed.

4.11. Duration of alcohol contents (alc_long): The total duration of all alcohol-related scenes combined in the MV?

*Only asked if (alc_image_bottle)=1 OR (alc_image_glass)=1 OR (alc_drinking)=1 OR (alc_drunk)=1*

1. <10 second

2. ≥10 seconds

4.12. Warning contents regarding drinking (alc_warn): The MV contains scenes that warn about drinking being restricted to individuals 18 years old and above [Y=1/N=0]

Important note: Any recognizable alcohol or tobacco content, even if blurry, was coded as such.

**Table 1: Definition and pictures of main smoking tobacco types and END in Vietnam**

| **Tobacco or ENDS** | **Definition** | **Pictures** |
| --- | --- | --- |
| Cigarette | A cigarette is a combination of cured and finely cut tobacco, reconstituted tobacco and other additives rolled or stuffed into a paper-wrapped cylinder | 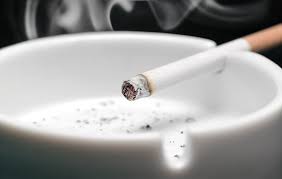 |
| Cigars | Cigars are tightly rolled bundles of fermented and dried tobacco leaves, typically larger, thicker than cigarettes and do not have a filter | 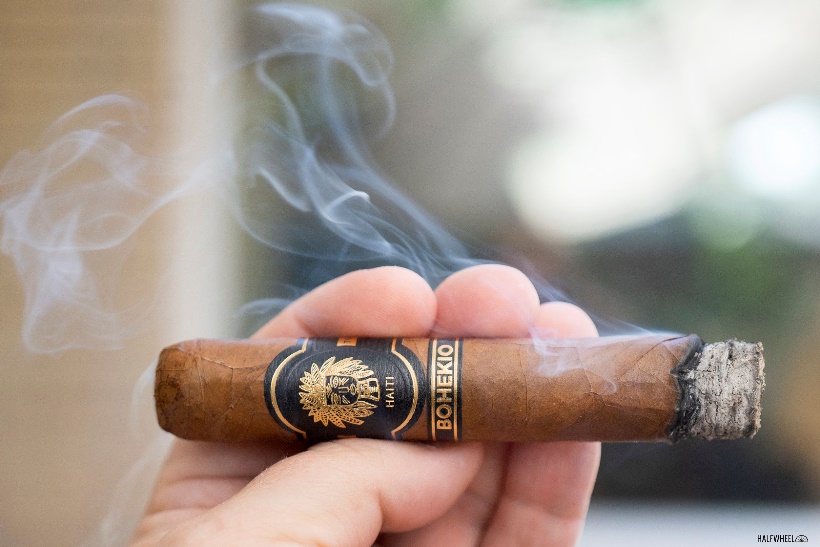 |
| Pipe tobacco | Pipes are often reusable and consist of a chamber or bowl, stem and mouthpiece. Tobacco is placed into the bowl and lit. The smoke is than drawn through the stem and mouthpiece and inhaled. | 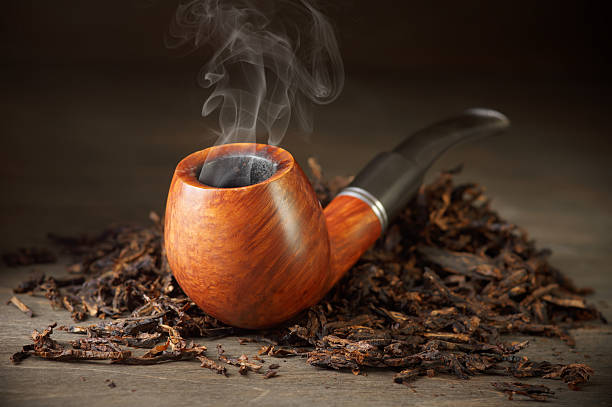 |
| Bamboo waterpipe tobacco | Smoking device made from bamboo, with a water filtration system, specifically designed for smoking tobacco. | 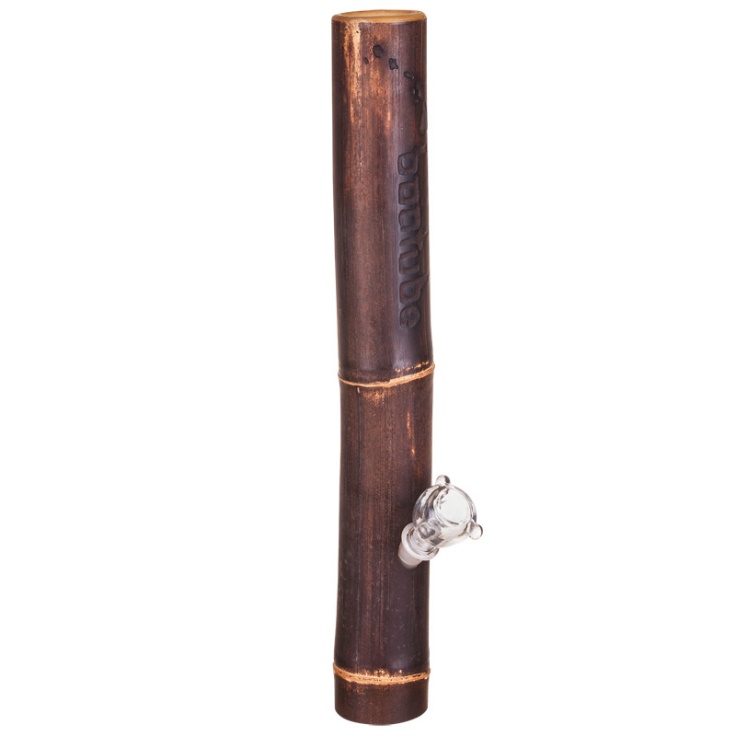 |
| Hookah | Hookah is a pipe used to smoke Shisha, a combination of tobacco and fruit or vegetable that is heated and the smoke is filtrated through water. The Hookah consists of a head, body water bowl and hose. The tobacco or Shisha is heated in the hookah usually using charcoal. | 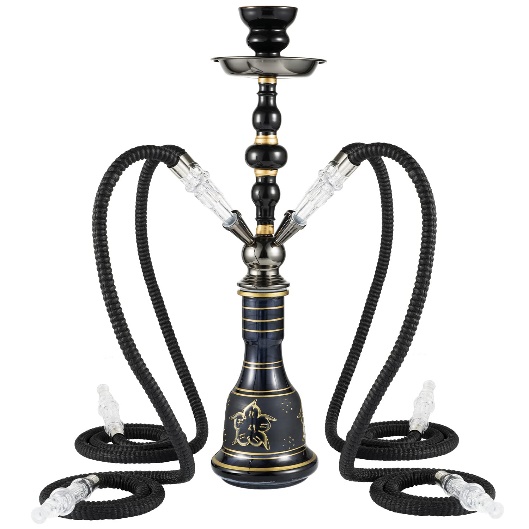 |
| E-cigarette | A device that has the shape of a cigarette, cigar, or pen and does not contain tobacco. It uses a battery and contains a solution of nicotine, flavorings, and other chemicals, some of which may be harmful. When electronic cigarettes are used, the nicotine solution turns into a mist that can be inhaled into the lungs. The amount of nicotine in individual e-cigarettes can vary. It is not yet known whether electronic cigarettes are safe or if they can be used to help smokers quit smoking. Also called e-cigarette. | 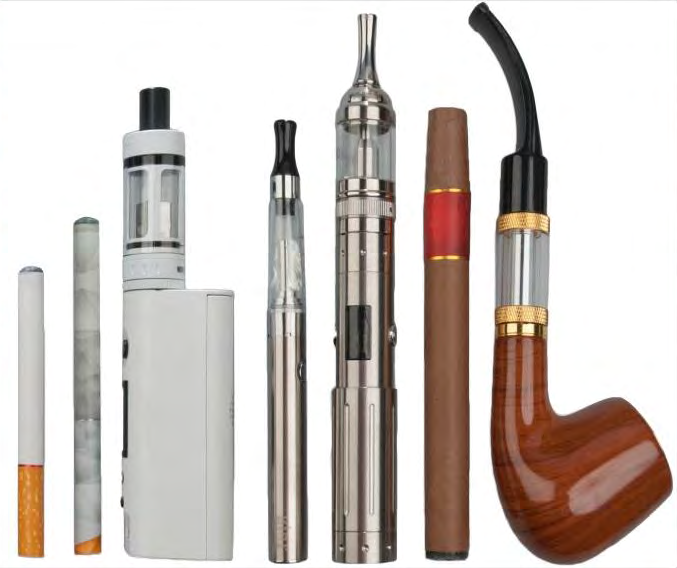 |
| Heated tobacco products | Tobacco products require the use of an electronic device to heat a stick or pod of compressed tobacco. The stick (by definition, a cigarette) or tobacco pod is heated to a temperature high enough to produce an inhalable aerosol, but the temperature is below that which is required for full combustion. | 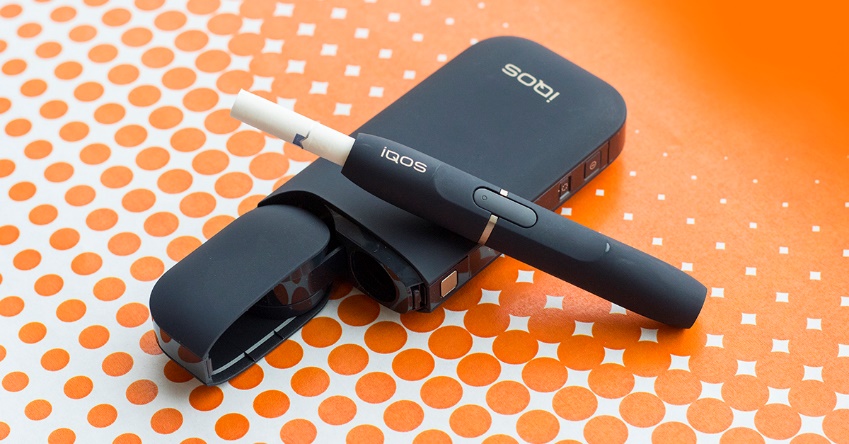 |
