# Supplementary figures and images for: Tobacco and Alcohol Content in Top Vietnamese YouTube Music Videos: Content Analysis
Source: J Med Internet Res. 2024 Nov 8;26:e55555. doi: 10.2196/55555 (PMC11584539; doi:10.2196/55555)

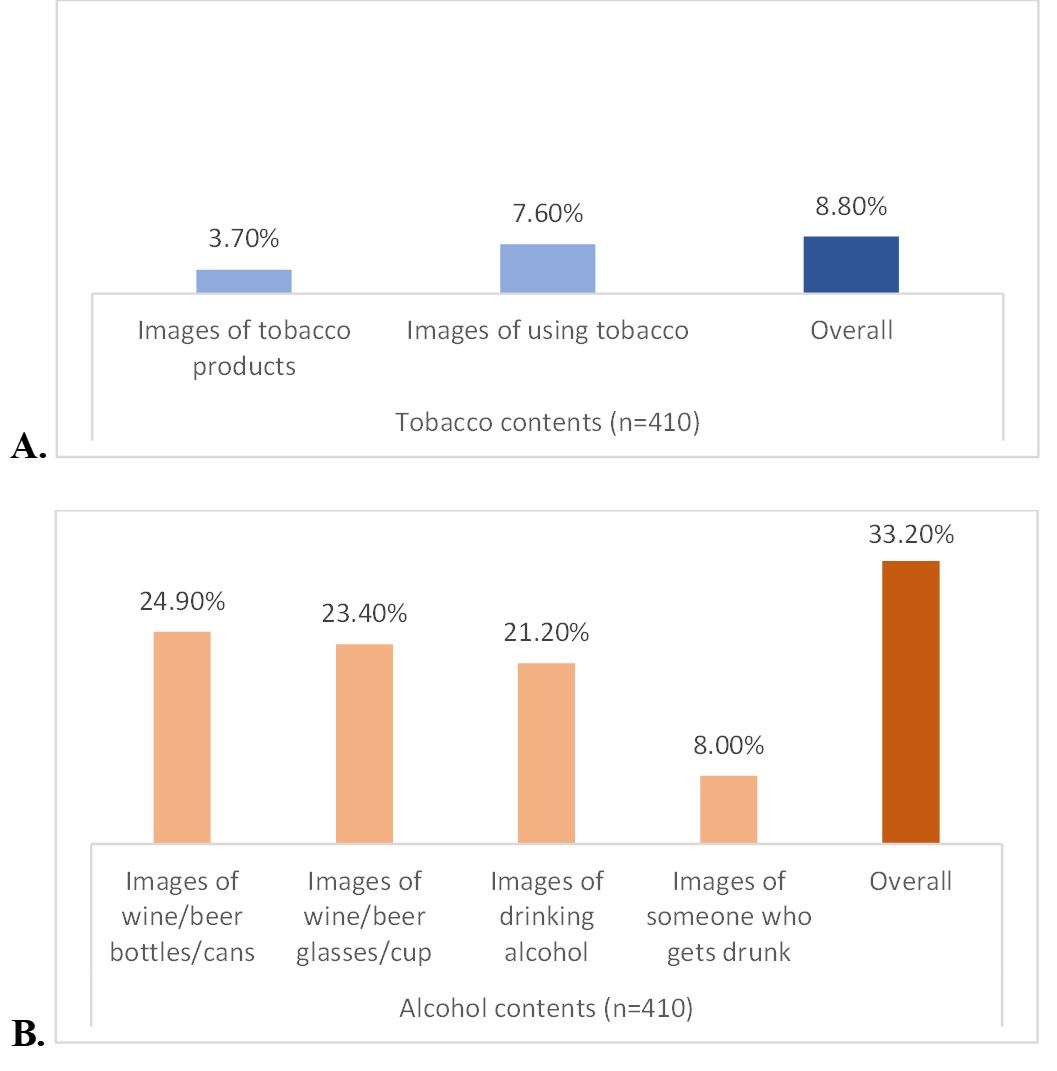

Supplement: Multimedia Appendix 3 [file jmir_v26i1e55555_app3.png]
